# Myelodysplastic syndrome-like response after voriconazole treatment of systemic lupus erythematosus complicated with fungal infection: a case report

**DOI:** 10.3389/fmed.2023.1286649

**Published:** 2023-12-07

**Authors:** Guang-Liang Xie, Xiao-Su Wang, Ling-Yan Hu, Yi Wang, Xiangchen Gu, Yan-Qiu Xu

**Affiliations:** ^1^Department of Nephrology, Yueyang Hospital of Integrated Traditional Chinese Medicine and Western Medicine, Shanghai University of Traditional Chinese Medicine, Shanghai, China; ^2^Department of Gastroenterology, Yueyang Hospital of Integrated Traditional Chinese Medicine and Western Medicine, Shanghai University of Traditional Chinese Medicine, Shanghai, China; ^3^Department of Hematology, Yueyang Hospital of Integrated Traditional Chinese Medicine and Western Medicine, Shanghai University of Traditional Chinese Medicine, Shanghai, China; ^4^Department of Nephrology, Ruijin Hospital, Shanghai Jiaotong University School of Medicine, Shanghai, China; ^5^Department of Medicine, Shanghai Hospital of Civil Aviation Administration of China, Shanghai, China

**Keywords:** voriconazole, myelodysplastic syndrome, hematocytopenia, adverse drug reaction, case report, systemic lupus erythematosus

## Abstract

**Background:**

Voriconazole is mainly used to treat progressive and potentially life-threatening infections in immunocompromised patients. The adverse drug reactions related to voriconazole are varied. In some rare cases, the use of voriconazole can result in myelodysplastic syndrome (MDS)-like adverse reactions.

**Case presentation:**

Here, we present a rare case of systemic lupus erythematosus patient with a fungal infection that developed MDS-like adverse reactions after treatment with voriconazole. The patient was admitted to the hospital because of 3 days of chest tightness and dyspnea. After the admission, the patient’s sputum culture showed *Candida albicans* infection, and voriconazole was prescribed to be taken orally. After using voriconazole, drug-related adverse reactions such as visual impairment, nausea, vomiting, hiccup, middle and lower abdominal pain, disorders of consciousness, delirium, hallucination, slow response, and subcutaneous ecchymosis appeared, as well as the gradually increased serum creatinine, oliguria, and aggravated lower limb edema. In addition, there was a decrease in peripheral blood cells, and MDS-like changes in bone marrow were indicated by bone marrow biopsy. After discontinuing voriconazole, drug-related adverse symptoms disappeared, and hematocytopenia and the changes in MDS were significantly improved, which was confirmed by a subsequent bone marrow puncture at a 6 months interval.

**Conclusion:**

This case reminded us that when using voriconazole for treatment, individual differences in patients should be considered, and the blood concentration of voriconazole should be closely monitored. Otherwise, potential drugs that affect voriconazole metabolism should be noted, and related adverse symptoms of patients should be closely observed during medication to reduce the occurrence of adverse drug events.

## Introduction

Voriconazole is primarily used to treat progressive and potentially life-threatening infections in immunocompromised patients. As a broad-spectrum triazole antifungal drug, voriconazole prevents the biosynthesis of ergosterol and produces antifungal effects by inhibiting the demethylation of 14a-sterols which is mediated by cytochrome P450 in fungi. In the human body, voriconazole is primarily metabolized in the liver while inhibiting the liver cytochrome P450 system. 80% of the metabolites are excreted in the urine, and 20% are excreted in the feces. This medication is efficient in treating invasive fungal *Aspergillus* and *Candida* infections. Most patients have a high tolerance for triazole antifungal medications. The most frequent side events, which are often mild to moderate, are vision impairment, fever, rash, nausea, vomiting, diarrhea, headache, hallucinations, peripheral edema, and abdominal discomfort (1–3). The most frequent side effects associated with medication withdrawal include liver failure, rash, and vision impairment. Additionally, gastrointestinal issues, such as nausea, abdominal pain, vomiting, and diarrhea, are the most often reported adverse events in clinical settings. The most frequent side effects of voriconazole were transient visual impairment (23% of patients), fever (12%), diarrhea (9%), and vomiting (7%) in a randomized, double-blinded, multicenter safety and tolerability study (4). The rate of adverse responses associated with voriconazole treatment was 18.5% (22/119), in another multicenter observational study evaluating the treatment of invasive aspergillosis in adults with voriconazole. Serious adverse reactions include hallucinations, toxic nephropathy, neurotoxicity, hepatotoxicity, psychogenic encephalopathy, liver side effects (10.1%), and nephrotoxicity (3.4%) (5). Moreover, the most recent voriconazole instruction manual refers to “rare adverse reactions” involving various systems, including myelodysplastic syndrome (MDS). However, clinical reports of voriconazole-related MDS-like reactions were rare. Herein, we report a case of a patient with systemic lupus erythematosus who had a fungal infection and experienced MDS-like following voriconazole therapy.

## Case presentation

A 73 years-old man was admitted to the hospital after experiencing dyspnea and chest tightness for 3 days. He has a 6 years history of gout, 5 years history of coronary heart disease, and a 2 years history of hypertension. He had long been treated for his disease with nifedipine GITS, clonidine, bisoprolol, and febuxostat. In addition, he received a diagnosis of systemic lupus erythematosus (SLE) due to skin erythema, photosensitivity, joint pain, positive for anti-dsDNA (228.65, normal <71 U/mL), anti-ANA (1.80), anti-Ro52 (99, normal <25), and decreases in complement C3 (0.574, normal 0.9–1.8 g/L) and C4 (0.088, normal 0.1–0.4 g/L) 2 years prior to admission and was taking prednisone 10 mg and hydroxychloroquine 100 mg daily, respectively. The patient’s laboratory results are displayed in Table 1.

**Table 1:** Laboratory results of the patient.

Laboratory test	On admission	3 weeks after voriconazole treatment	2 weeks after voriconazole discontinuation
Hemoglobin (g/L)	84	48	71
WBC (×10^9^/L)	2.4	1.4	3.9
Platelet (×10^9^/L)	73	8	69
C-reactive protein (mg/L)	6.66	5.03	0.97
Creatinine (μmol/L)	290.0	580.1	218.8
Blood urea nitroge (mmol/L)	28.4	65.7	25.5
Uric acid (μmol/L)	405.5	421.0	366.2
ALT (U/L)	28	21	15
AST (U/L)	25	2	7
r-GT (U/L)	58	78	38.1
Anti-dsDNA (U/mL)	42	13	13
Complement C3 (g/L)	0.87	0.92	0.85
Anti-ANA	Negative	Negative	Negative

After admission, the pulmonary computer tomography (CT) scan was performed, and the results revealed pulmonary interstitial changes, pulmonary edema, and a small amount of pleural effusion on both sides, and his blood oxygen saturation was 75% (93% after oxygen inhalation), and the pulmonary infection was treated with cefminox sodium, piperacillin/tazobactam, and meropenem successively. Otherwise, a total of 120 grams of human immunoglobulin were administered to help improve the immune system of the patient.

**Figure 1:**
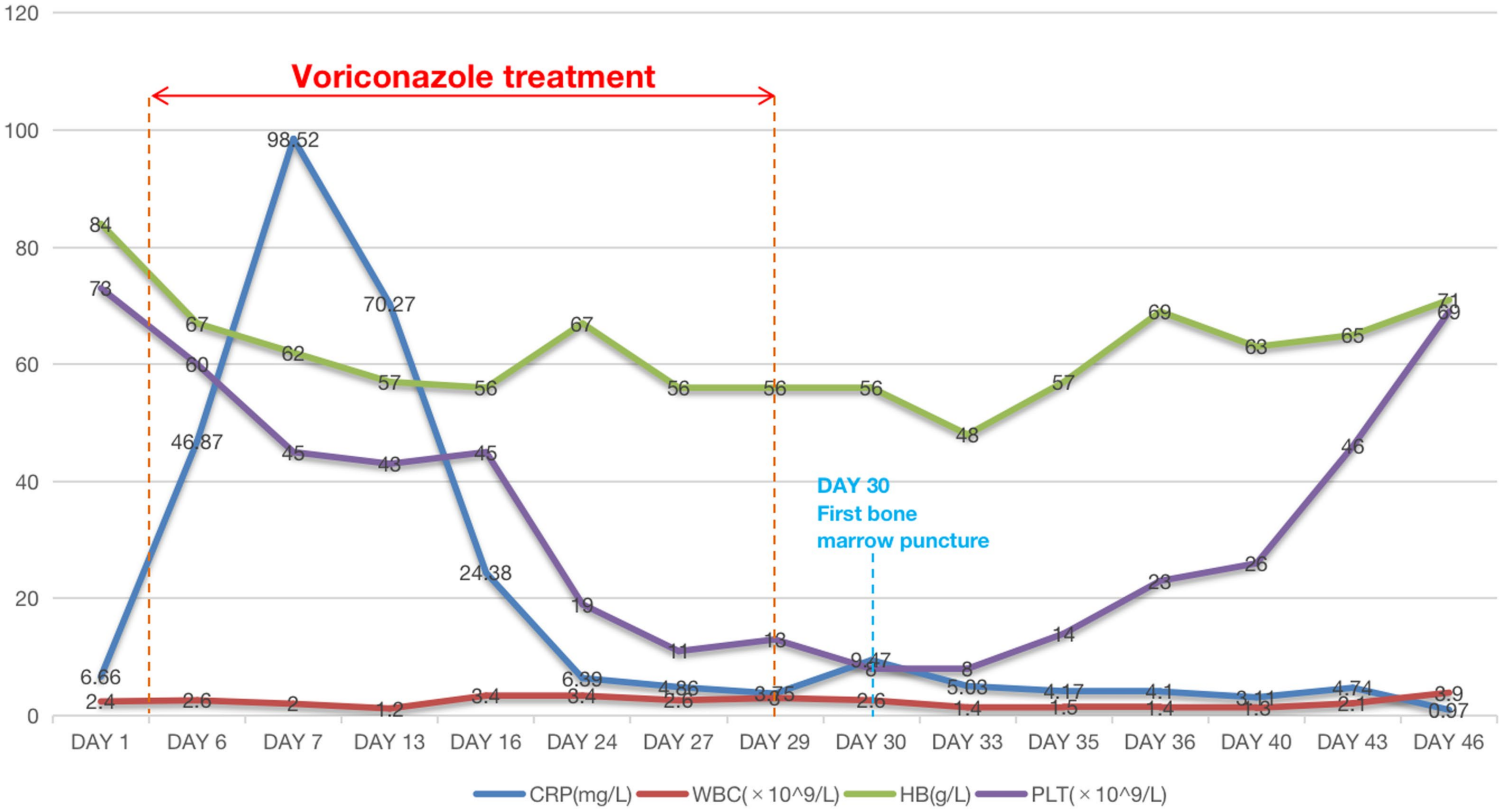
Changes in patient laboratory test results.

The patient was given oral voriconazole (200 mg/day) for 25 days after the patient’s sputum culture revealed *Candida albicans* infection and the serum (1,3)-β-D-glucan (+) (542 pg/mL, normal <60 pg/mL). Following the administration of voriconazole, we noticed a progressive decline in the patient’s hemoglobin, leukocyte, and platelet counts in the patient’s CBC test (Figure 1), accompanied by symptoms including visual impairment，nausea, vomiting, hiccups, middle and lower abdominal pain, disorders of consciousness, delirium, hallucination, slow response, and subcutaneous ecchymosis. AKI diagnosis criteria were also met by the patient’s oliguria, gradually rising serum creatinine level, aggravated pleural effusion, and lower limb edema. The patient was treated with hemodialysis and intravenous methylprednisolone (40 mg) daily. When renal function, heart failure, pleural effusion, and edema rapidly improved and urine volume returned to above 1,000 mL per day, hemodialysis was discontinued. The patient’s hemoglobin, leukocyte, and platelets gradually increased once the voriconazole was discontinued. At a follow-up of 14 days, we observed that these markers continued to improve.

**Figure 2:**
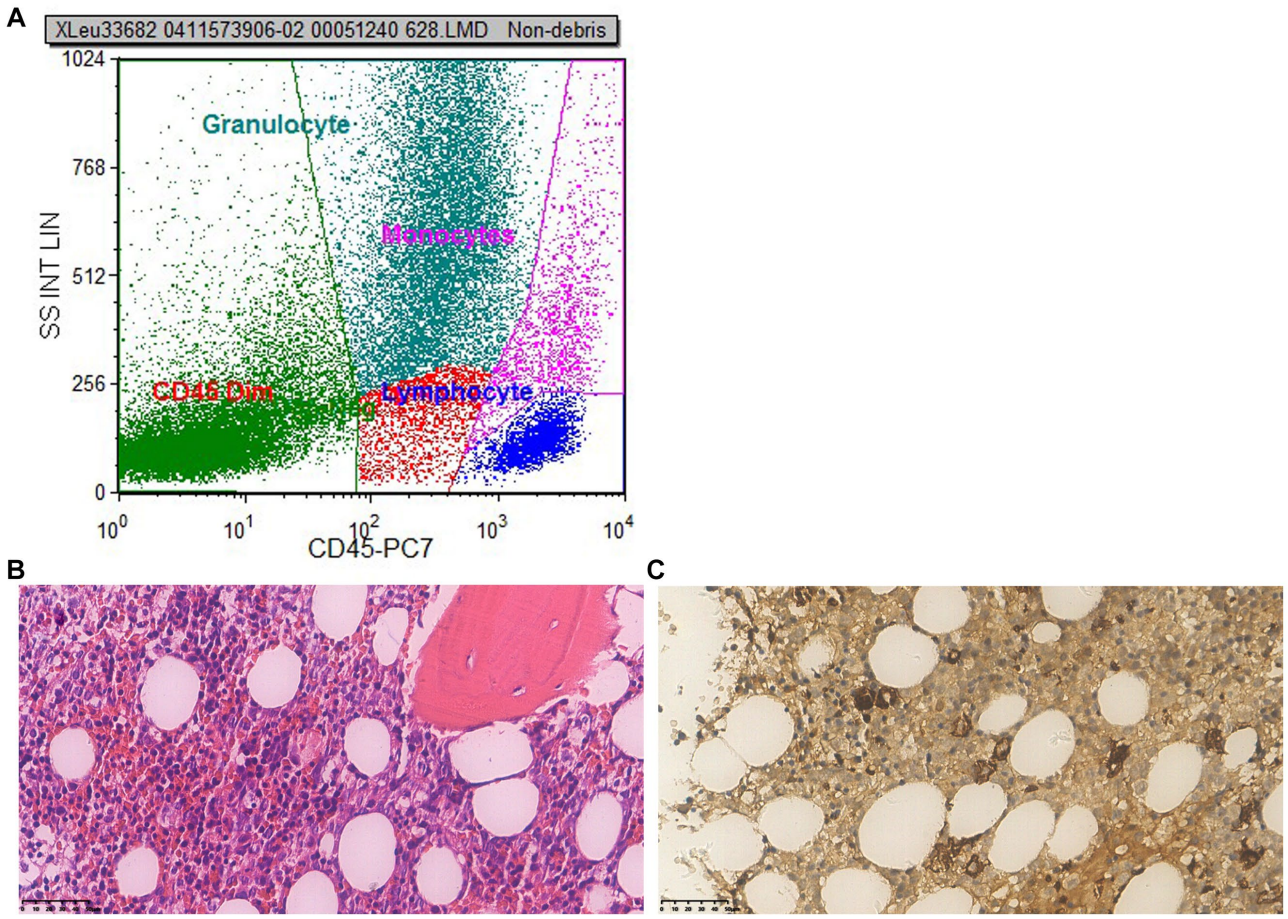
First bone marrow puncture results. **(A)** Flow cytometry: flow cytometry shows 1.6% of myeloid cells were primitive or immature. **(B)** Microscope (×400): the proliferation of nucleated red blood cells in the bone marrow is active, and primitive/immature cells are readily visible; the amount of erythrocytes increases, megaloblastic cells are easy to find; megakaryocytes are not decreased, no obvious collagen fibrosis in the bone marrow stroma. **(C)** Immunohistochemistry (×400): CD34 primitive/immature cells (+), accounting for 10%–15%, some of which are weak positive and tend to be erythroid precursor cells; megakaryocyte (+), small megakaryocytes and lymphoid small megakaryocytes are easily seen; MPO granulocyte (+); CD71 nucleated red blood cells (+); a small amount of CD20 B cells are scattered (+); CK (−); a small amount of NK cells in CD56 are scattered (+). Conclusion: myelodysplastic syndrome (MDS) should be taken into consideration, the erythroid hyperplasia is significantly active with megaloblastic transformation.

During the course of the patient’s treatment, red blood cells (600 mL), fresh frozen plasma (800 mL), and platelet concentrate (400 mL) were transfused. The blood alterations are depicted in Figure 1, but there was no discernible improvement in stopping the decreasing trend of blood cell count. We performed a bone marrow biopsy the day after discontinuing voriconazole to rule out any potential pathology that might cause hematopenia. Bone marrow aspirate showed active bone marrow hyperplasia. Nucleated cells proliferated actively, and the ratio of granulocytes to red blood cells was 0.387:1. Granular proliferation decreased, accounting for 27.5% of nuclear cells, with primitive cells accounting for 4.5%, the proliferation was mainly characterized by neutrophils, late promyelocytes, rod-shaped nuclei, and lobulated nuclei, with no abnormalities in morphology or size. Erythrocytosis was extremely active, accounting for 71% of nuclear cells, among them, primary red blood accounts for 5.5%, mainly consisting of proliferation of middle and late erythroblasts, imbalance in nuclear and cytoplasmic development, nuclear abnormalities, and megaloblastic transformation of erythroblasts could be observed, the size of mature red blood cells varies significantly, pathological hematopoiesis was very obvious. Megakaryocyte proliferation is very active with maturation disorders. Iron staining: external iron: 2 (+), internal iron: 80% iron granulosa erythrocytes. In addition, genetic testing was negative. Fluorescence *in situ* hybridization (FISH) was used to perform genetic testing on bone marrow aspirates and did not find any chromosomal abnormalities or gene deletions, including –7/7q–, +8, p53 (17p13.1), EGR1 (5q31), D20S108, etc. Flow cytometry detection of bone marrow (Figure 2A) showed that 1.6% of myeloid cells were primitive or immature. Microscope (Figures 2B,C) indicated that myelodysplastic syndrome (MDS) should be considered, erythroid hyperplasia is significantly active with megaloblastic transformation.

**Figure 3:**
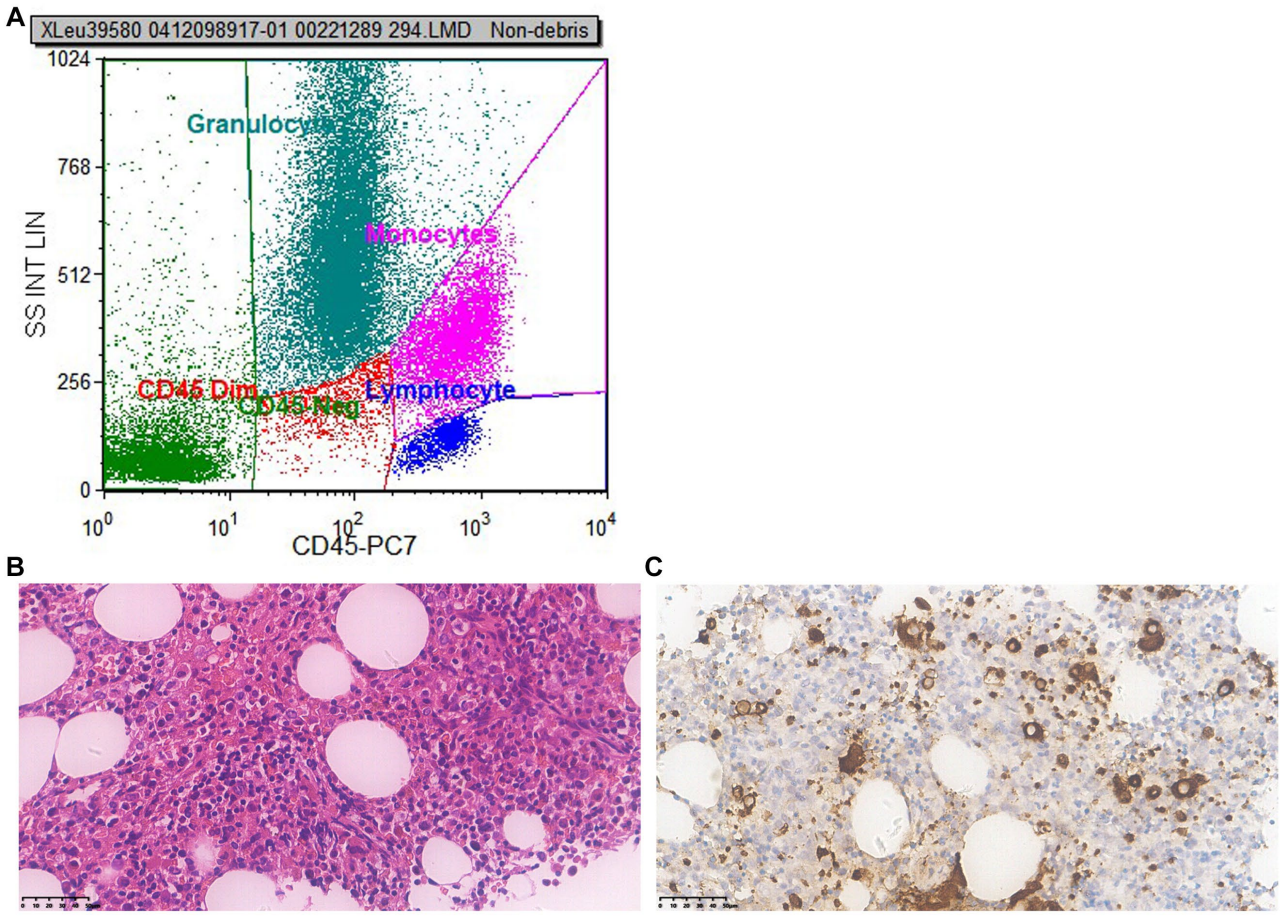
Second bone marrow puncture results 6 months later. **(A)** Flow cytometry: flow cytometry shows no significant evidence of acute leukemia, NHL, or high-risk MDS-related immunophenotypic abnormalities were detected. **(B)** Microscope (×400): the proliferation of nucleated red blood cells in the bone marrow is active; cells in all stages of the granulocyte lineage are visible, with the majority being cells in the more mature stage, while pale nucleus-like cells are easily visible; the number of red blood cells is small, mainly composed of middle to late-stage erythrocytes distributed in scattered and small clusters; the number of recognizable megakaryocytes is small, with varying sizes and scattered distribution; no obvious collagen fibrosis was observed between the bone trabeculae. **(C)** Immunohistochemistry (×400): CD34 primitive/immature cells (+), accounting for about 1%; CD117 primitive/immature cells (+), no increase observed; CD42b: megakaryocyte (+), increased in number, mainly small megakaryocytes; CK (−); CD71 nucleated red blood cells (+); MPO granulocyte (+); CD138 A few plasma cell were scattered (+). Conclusion: bone marrow test was negative, no indication of MDS.

To ascertain whether the patient had fully recovered, we conducted another bone marrow puncture after a 6 months follow-up. The results of the second bone marrow aspiration revealed a significant reduction in the abnormal proliferation of erythroid, granulocytic, and megakaryocytic cells compared to the first, and no obvious MDS-like bone marrow abnormal proliferation was discovered. Flow cytometry results (Figure 3A) demonstrated that no significant evidence of acute leukemia, NHL, or high-risk MDS-related immunophenotypic abnormalities were detected. Microscopic results bone marrow test was negative, no indication of MDS (Figures 3B,C).

Overall, when treating the patient with voriconazole during the first-hospital admission, we observed decreases in peripheral blood cells and diagnosed MDS-like changes in bone marrow by bone marrow puncture. After voriconazole withdrawal, drug-related side effects vanished, and the MDS alterations were significantly improved.

## Discussion

The metabolism of voriconazole and the symptoms of adverse medication reactions differ dramatically between different races and individuals. Voriconazole metabolism *in vivo* exhibits a nonlinear pharmacokinetics characteristic, meaning that when the dosage is increased, the area under the drug time curve (AUC) increases sharply. The steady blood concentration was reached in about 5 days after intravenous or oral administration (6). In addition to being metabolized by liver cytochrome P450 Isozyme CYP2C19, CYP2C9, and CYP3A4, Voriconazole also inhibits these three enzymes. The pharmacokinetics of voriconazole vary significantly among individuals. *In vivo* studies have revealed that the main metabolic pathway, CYP2C19, exhibits genetic polymorphism with individual variations of up to 100 times. The population can be categorized into individuals with strong metabolism and individuals with weak metabolism based on the activity of this enzyme. While the prevalence of individuals with poor metabolisms ranges from 3% to 5% among Caucasians and African Americans, it can reach up to 15%–20% among Asians. In China, the phenotypes of people with weak CYP2C19 metabolism are nearly CYP2C19 * 2 and CYP2C19 * 3. According to research studies in healthy Caucasians and Japanese, the concentration of voriconazole in individuals with weaker metabolism within the same race was on average, four times greater in those with better metabolisms. The primary plasma metabolite of voriconazole is N-oxide, which accounts for approximately 72% of the total plasma. The half-life of voriconazole is around 6 h, and its distribution volume is 2–4.6 L/kg, indicating that it is broadly distributed in tissues. The genotype of CYP2C19 and the co-administration of medications that regulate the activity of CYP2C19, CYP2C9, and CYP3A4 could all have an impact on the blood concentration of voriconazole. The metabolism of CYP substrates may also be inhibited by voriconazole (7–10).

In this case, after taking voriconazole, this patient experienced symptoms of psychiatric encephalopathy, nausea, vomiting, hiccup, middle and lower abdominal pain, diarrhea, delirium, hallucinations, delayed response, and clinical signs such as progressive elevation of creatinine and renal toxicity, even blood cells counts and bone marrow also changed. These symptoms are consistent with the side effects listed in the instructions and early clinical studies. However, the majority of the side effects reported in previous clinical reports typically coexist with a few symptoms in a single patient. Nevertheless, in this case, the patient’s use of voriconazole led to more complicated symptoms mentioned above, indicating the patient’s susceptibility and intolerance to voriconazole’s side effects.

It is important to highlight that this patient had a 2 years history of SLE. The phenomenon of SLE combined with hematological diseases has been widely reported in China. However, cases of combining with MDS are rare (11–13). The mechanism of bone marrow morphological changes in SLE patients is not yet elucidated. Some have suggested there are various antibodies against blood cells in the serum of SLE patients, which directly damage a certain lineage of hematopoietic progenitor cells or alter the bone marrow microenvironment, causing bone marrow dysplasia in SLE patients (14). MDS, which frequently arises during the active phase of SLE, has been linked to SLE in some studies (15, 16). Meanwhile, the patient was diagnosed with SLE for 2 years, but, there had been no significant reduction in his blood cells while receiving long-term steroid medication. In addition, there was no obvious increase in the levels of ANA, dsDNA antibody, rheumatoid factor, or decrease in C3 in the patient, and there was no joint swelling and pain, nor were there any other SLE symptoms like new erythra, indicating relevant performance during SLE flares. Although C-reactive protein (CRP) is also an indicator of SLE activity, the patient’s CRP showed a transient spike. The increase in CRP was accompanied by exacerbation of pulmonary infection, indicating infection rather than SLE flare. It was unlikely related to SLE activity since it immediately returned to normal after the anti-infection and antifungal treatment. Based on the aforementioned findings, SLE activity and SLE-related triple decline or SLE-related MDS can be essentially ruled out.

We also took into account whether the patient developed primary MDS. MDS is a group of heterogeneous myeloid clonal diseases originating from hematopoietic stem cells, characterized by abnormal differentiation and development of myeloid cells, manifested as ineffective hematopoiesis, refractory hemocytopenia, hematopoietic failure, and high-risk transformation to acute myeloid leukemia (AML) (17, 18). The following conditions must be met for the MDS diagnosis: (1) continuous (≥6 months) reduction of one or more lineages of blood cells: red blood cells (Hb <110 g/L); neutrophils (ANC < 1.5 × 10^9^/L); platelets (BPC <100 × 10^9^/L). (2) Exclude other hematopoietic and non-hematopoietic system diseases that can lead to reduced blood cells and pathological hematopoiesis. Determination criteria: (1) morbid hematopoiesis: bone marrow smear with at least 10% of any lines of red blood cell, neutrophil, or megakaryocyte; (2) the proportion of circular iron granulocytes occupying nuclear red blood cells is ≥15%; (3) primitive cells: 5%–19% in bone marrow smear; (4) chromosome abnormalities (19, 20). The literature indicates that MDS has multiple gene mutations, and spontaneous self-remission in *de novo* MDS is very rare (21, 22). The results of the patient’s first bone marrow biopsy revealed MDS-like characteristics. Interestingly, the hemocytopenia resolved spontaneously after discontinuation of voriconazole and without any anti-MDS treatment. The bone marrow biopsy examined 6 months later revealed a notable improvement, indicating no MDS-like disease. Besides, no gene mutations were found in the two bone marrow biopsies with a 6 months interval before and after. Taken together, these findings rule out primary MDS, and conform to the characteristics of drug-related MDS.

The possibility of additional medications causing hemocytopenia during the treatment should also be taken into consideration. In the treatment of pulmonary infection, in addition to voriconazole, we also used cefminox sodium (4 days), piperacillin/tazobactam (3 days), and meropenem (29 days) successively for anti-infection. According to the instructions for these drugs, the side effect of cefminox sodium is occasional pancytopenia (23); side effects of piperacillin/tazobactam include leukopenia, neutropenia, thrombocytopenia and other side effects (rare)，whole blood cell reduction (very rare) (24–26); side effects of meropenem include pancytopenia, agranulocytosis, hemolytic anemia (frequency unknown), leukopenia, thrombocytopenia (<1%) (27). The first two drugs have been used for a short period of time (only 3 and 4 days, respectively), and the incidence of side effects caused by drug-related hemocytopenia and MDS-like change in bone marrow is very low. Meropenem basically covered most of the course of inpatient treatment; among the three drugs, it seems to have the highest likelihood of causing the patients’ hemocytopenia. Meropenem is a kind of carbapenem antibiotic, and carbapenem antibiotics usually have the characteristics of a broad spectrum and strong effect. The side effects of meropenem mainly include the following aspects: first, it may be prone to allergic reactions, such as skin erythema, itching, fever, redness, etc. In addition, it may have an impact on the blood system, and some patients may experience a decrease in granulocytes or platelets after use or may also experience an increase. However, the blood cells of the patient had decreased significantly before the use of meropenem, and the number of blood cells began to recover during the treatment without interrupting the use of it. Therefore, the evidence of hemocytopenia caused by meropenem is insufficient.

Figure 1 illustrates the patient’s decreased blood cell count upon arrival. Following the discontinuation of voriconazole, the patient’s blood cell count gradually increased but did not reach its pre-voriconazole values. The patient has a long-term history of SLE, as was already indicated, which may contribute to a decrease in blood cell count. The patient’s condition, though, remained largely constant, and there had never before been an abrupt drop in blood cells. Following the administration of voriconazole, the patient’s blood cell count dropped quickly and steadily, with platelets experiencing the greatest decline, dropping to a minimum of 8.0 × 10^9^/L. After discontinuing voriconazole, the patient’s blood cell count steadily improved until it reached the pre-disease level, though it was still lower than that of healthy individuals. Two possible explanations include the following: first, even though discontinuing voriconazole, the drug residue in the body was not immediately metabolized, and the drug side effects still played a role to some extent; secondly, although the patient’s SLE condition remained stable, however, SLE continues to exist and will not disappear, and the objective phenomenon of cell reduction caused by it still exists.

The limitation of this case report is that we did not monitor the blood concentration of voriconazole to rule out the possibility of drug overdose. It is also important to understand the exact mechanism of voriconazole or its metabolites-induced bone marrow suppression in future studies.

## Conclusion

Voriconazole was the cause of the patient’s MDS-like bone marrow suppression. This case report reminds us that in clinical practice, when using voriconazole for anti-fungal infection treatment, individual differences in patients should be taken into consideration, and the serum concentration of voriconazole should be closely monitored. On the other hand, potential medications that affect voriconazole metabolism should also be noted, and patients should be carefully observed during medication to avoid the occurrence of adverse drug events.

## Data availability statement

The original contributions presented in the study are included in the article/supplementary material, further inquiries can be directed to the corresponding authors.

## Ethics statement

Ethical approval was not required for the studies involving humans because this is a retrospective case report, written informed consent was obtained from the individual(s) for the publication of any potentially identifiable images or data included in this article. The studies were conducted in accordance with the local legislation and institutional requirements. The human samples used in this study were acquired from this is a retrospective case report, all the data and images are based on previous medical treatment and examination results, and written informed consent was obtained from the individual(s) for the publication of any potentially identifiable images or data included in this article.

## Author contributions

G-LX: Conceptualization, Formal analysis, Writing – original draft, Investigation. X-SW: Data curation, Investigation, writing – review & editing. L-YH: Data curation, Formal analysis, Writing – original draft. YW: Investigation, Writing – original draft. XG: Data curation, Project administration, Writing – review & editing. Y-QX: Methodology, Project administration, Resources, Writing – review & editing.
